# Reply: To See the Telemedicine Forest for Its Multicultural Trees. Comment on: In‐Home Remote Assessment of the MDS‐UPDRS Part III: Multi‐Cultural Development and Validation of a Guide for Patients

**DOI:** 10.1002/mdc3.70072

**Published:** 2025-05-13

**Authors:** Michelle H.S. Tosin, Alvaro Sanchez‐Ferro, Ruey‐Meei Wu, Beatriz G.R.B. de Oliveira, Marco Antonio A. Leite, Pablo Rábano Suárez, Christopher G. Goetz, Pablo Martinez‐Martin, Glenn T. Stebbins, Tiago A. Mestre

**Affiliations:** ^1^ Department of Neurological Sciences Rush University Medical Center Chicago Illinois USA; ^2^ Department of Nursing Fluminense Federal University Rio de Janeiro Brazil; ^3^ Hospital Universitario Madrid Spain; ^4^ Department of Neurology National Taiwan University Hospital, College of Medicine, National Taiwan University Taipei Taiwan; ^5^ Department of Clinical Medicine, Postgraduate Program in Neurology and Neurosciences Fluminense Federal University Rio de Janeiro Brazil; ^6^ Parkinson's Disease and Movement Disorders Center, Division of Neurology, Department of Medicine, The Ottawa Hospital Research Institute University of Ottawa Brain and Mind Institute Ottawa Ontario Canada

**Keywords:** MDS‐UPDRS, Parkinson's disease, telemedicine, video guide

We appreciate the letter from Goraieb et al.^1^ that shares the results of their study evaluating telemedicine use in Brazil and the various educational, social, and economic factors that may impact its use.^1^ The article's findings highlight the potential of telemedicine in movement disorders and how the “In‐Home Remote Assessment of the MDS‐UPDRS Part III” can enhance its utilization.

We developed a culturally sensitive, quasi‐universal guide for the Movement Disorder Society‐Unified Parkinson's Disease Rating Scale (MDS‐UPDRS) part III in‐home remote assessment that can be used in as many regions of the globe as possible.

Goraieb et al.^1^ highlight that telemedicine offers significant cost savings, including reducing transportation and meal expenses and indirect savings related to caregiving. These financial benefits can help alleviate the burden associated with traditional in‐person consultations. However, it is crucial to recognize that, depending on the social‐cultural context, patients with Parkinson's disease (PD) may still prefer face‐to‐face medical visits,^2^ as observed at our Taiwan site. Additionally, telemedicine may not be suitable for managing all movement disorders.^3^

Beyond resource limitations, telemedicine can offer flexibility for patients and clinicians who choose it as a means of care. Personalized care decisions based on a direct view of the patient's home environment is a particular feature of this technology. Telemedicine's adaptability is particularly relevant in some countries, where potential restrictions on telemedicine coverage may impact access to remote care^4^ and prevent a portion of the population from accessing quality and standardized healthcare.

Apart from these considerations, we hope the MDS‐UPDRS Part III In‐Home Remote Assessment Guide, available through the MDS, becomes a valuable tool for optimizing global care delivery in PD. By providing standardized guidance to patients when being examined remotely, the guide strengthens telemedicine's role in bridging healthcare gaps and enhancing patient outcomes, aligning with the MDS's mission.

## Author Roles

(1) Research project: A. Conception, B. Organization, C. Execution; (2) Statistical Analysis: A. Design, B. Execution, C. Review and Critique; (3) Manuscript: A. Writing of the First Draft, B. Review and Critique.

M.H.S.T.: 1A, 1B, 1C, 3A

A.S.F.: 3B

R.M.W.U.: 3B

B.G.R.B.O.: 3B

M.M.M.L.: 3B

P.R.S.: 3B

C.G.G.: 3B

P.M.M.: 3B

G.T.S.: 3B

T.A.M.: 1A, 1B, 1C, 3A

## Disclosures

**Ethical Compliance Statement**: The authors confirm that the approval of an institutional review board was not required for this work. Informed consent is not applicable. We confirm that we have read the Journal's position on issues involved in ethical publication and affirm that this work complies with its guidelines.

**Funding Sources and Conflict of Interest**: No specific funding was received for this work. The authors declare no conflicts of interest relevant to this work.

**Financial Disclosures for the previous 12 months**: M.H.S.T. Salary—Rush University; Professional Societies: working group supported by the International Parkinson and Movement Disorder Society. A.S.F. received grants or contracts from ERA‐NET Horizon 2020 program JPCOFUND2 (reference number HESOCARE‐329‐073) and Instituto de Salud Carlos III (reference number P122/01177); consulting fees from AbbVie, Boston Scientific, ESTEVE, Orion Pharma, Prim and Roche; and payment or honoraria for lectures, presentations, speakers bureaus, manuscript writing, or educational events from AbbVie, Bayer, ESTEVE, MDS Society, EAN, Novartis, Monitor, Organon, Roche, SEN, Stada, Teva, and Zambon. R.M.W.U. received a grant for research from the National Science and Technology Council of Taiwan; a salary from the National Taiwan University Hospital and National Taiwan University; and received honoraria from the International Parkinson and Movement Disorder Society for activities during the International Congress 2022 and 2023. B.G.R.B.O. received grants and research from the National Council for Scientific and Technological Development (CNPq) and the Research Support Foundation of the State of Rio de Janeiro (FAPERJ) and salary from Fluminense Federal University. M.M.M.L. received salary from Fluminense Federal University. P.R.S. has received speaker honoraria from Bial, Ipsen, Stada, and Zambon and research grants from the MDS Society (e‐Diary Project). C.G.G. received consulting or Advisory Board Membership with honoraria from Genentech, Psychogenics, CSG Health Group, and CHDI; received grants/research funding to Rush University Medical Center from NIH, Department of Defense, UO1 funding from NIDDK, the International Parkinson and Movement Disorder Society, and The Michael J. Fox Foundation for research conducted by C.G.G.; received honoraria from the Faculty and Program Management stipend from the International Parkinson and Movement Disorder Society and Volume Editor stipend from Elsevier Publishers; received royalties from Elsevier Publishers, Wolters Kluwer, and Oxford University Press; received salary from Rush University Medical Center. P.M.M. has nothing to declare. G.T.S. reports consulting and advisory board membership with honoraria from Acadia, Pharmaceuticals, Adamas Pharmaceuticals, Biogen, Ceregene, CHDI Management, Cleveland Clinic Foundation, Ingenix Pharmaceutical Services (i3 Research), MedGenesis Therapeutix, Neurocrine Biosciences, Pfizer, Tools‐4‐Patients, Ultragenyx, and the Sunshine Care Foundation; received grants and research from the National Institutes of Health, Department of Defense, The Michael J. Fox Foundation for Parkinson's Research, Dystonia Coalition, CHDI, Cleveland Clinic Foundation, International Parkinson and Movement Disorder Society, and CBD Solutions. G.T.S. reports honoraria from the International Parkinson and Movement Disorder Society, the American Academy of Neurology, The Michael J. Fox Foundation for Parkinson's Research, the Food and Drug Administration, the National Institutes of Health, and the Alzheimer's Association; received a salary from Rush University Medical Center. T.A.M. had compensations for consulting and being an Advisory Board member for the following organizations: AbbVie, International Parkinson and Movement Disorder Society, CHDI Foundation/Management, Sunovion, Merz, Roche, PTC therapeutics; received research grants from the following organizations: EU Joint Program–Neurodegenerative Disease Research, uOBMRI, Ontario Research Fund, the Canadian Institutes of Health Research, The Michael J. Fox Foundation, Parkinson Canada, Parkinson Research Consortium, and Brain Canada.
